# Effects of maternal characteristics and medical history on first trimester biomarkers for preeclampsia

**DOI:** 10.3389/fmed.2023.1050923

**Published:** 2023-01-24

**Authors:** Johnatan Torres-Torres, Salvador Espino-y-Sosa, Jose Rafael Villafan-Bernal, Luis Enrique Orozco-Guzman, Juan Mario Solis-Paredes, Guadalupe Estrada-Gutierrez, Romeo Adalid Martinez-Cisneros, Paloma Mateu-Rogell, Sandra Acevedo-Gallegos, Raigam Jafet Martinez-Portilla

**Affiliations:** ^1^Clinical Research Branch, Instituto Nacional de Perinatología Isidro Espinosa de los Reyes, Mexico City, Mexico; ^2^Iberoamerican Research Network in Obstetrics, Gynecology, and Translational Medicine, Mexico City, Mexico; ^3^Laboratory of Immunogenomics and Metabolic Diseases, Instituto Nacional de Medicina Genómica (INMEGEN), Mexico City, Mexico; ^4^Servicios de Salud Pública de La Ciudad de México, Mexico City, Mexico

**Keywords:** preeclampsia, multiples of the median (MoMs), first trimester prediction, Latin America, biomarkers

## Abstract

**Objective:**

To identify and quantify the effects of maternal characteristics and medical history on the distribution of Placental Growth Factor (PlGF), mean arterial pressure (MAP), and Uterine Artery Mean Pulsatility Index (UtA-PI); and to standardize the expected values for these biomarkers in the first trimester to create unique multiples of the median (MoMs) for Latin-American population.

**Methods:**

This is a prospective cohort built exclusively for research purposes of consecutive pregnant women attending their first-trimester screening ultrasound at a primary care center for the general population in Mexico City between April 2019 and October 2021. We excluded fetuses with chromosomal abnormalities, major fetal malformations, and women delivering in another care center. Linear regression was used on log-transformed biomarkers to assess the influence of maternal characteristics on non-preeclamptic women to create MoM.

**Results:**

Of a total of 2,820 pregnant women included in the final analysis, 118 (4.18%) developed PE, of which 22 (0.78%) delivered before 34 weeks of gestation, 74 (2.62%) before 37 weeks, and 44 (1.56%) from 37 weeks gestation. Characteristics that significantly influenced PLGF were fetal crown rump length (CRL), maternal age, nulliparity, body mass index (BMI), chronic hypertension, Lupus, spontaneous pregnancy, polycystic ovary syndrome (PCOS), hypothyroidism, preeclampsia (PE) in a previous pregnancy, and mother with PE. MAP had significant influence from CRL, maternal age, PE in a previous pregnancy, induction of ovulation, a mother with PE, chronic hypertension, BMI, and hypothyroidism. UtA-PI was influenced by CRL, maternal age, a mother with PE, chronic hypertension, and gestational diabetes mellitus (GDM) in a previous pregnancy.

**Conclusion:**

Population-specific multiples of the median (MoMs) for PlGF, MAP, and UtA-PI in the first trimester adequately discriminate among women developing preeclampsia later in pregnancy.

## Introduction

Preeclampsia (PE) is the result of an angiogenic disbalance (1), which remains one of the leading causes of maternal mortality and a public health challenge affecting 3–5% of all pregnancies (2, 3). Despite the huge amount of research regarding the prevention of preeclampsia, multivariate models have not been implemented worldwide, and Aspirin has not been adopted as a standard of care for all women at higher risk, especially in developing countries. Several prediction models have been created for the selection of patients at risk, achieving up to 82% of detection rate (4, 5). The most used models are the one from the Fetal Medicine Foundation, which uses a survival analysis called the competing risks model (6) for the prediction of women developing the diseases, while another widely known model comes from the Hospital Clinic of Barcelona that uses a Bayesian analysis under a logistic model (4). Although both models use different statistical techniques, they comprise a multiple analysis of maternal history and biomarkers such as uterine artery Doppler (UtA), placental growth factor (PlGF), and blood pressure, which have demonstrated better detection rates compared to maternal history alone (5, 7), however, biomarkers are still far from being robust enough to adequately discriminate those women developing preeclampsia, mainly due to the lack of robust methodology in their measurement and analysis (8). The ultimate goal for the detection of women at risk of developing preeclampsia is the prevention of the disease by using Aspirin before 16 weeks which has demonstrated in randomized trials a reduction of 62% of preeclampsia before 37 weeks of gestation and 82% before 34 weeks (9). However, there have been several attempts to validate FMF’s model in different populations failing to even achieve similar detection rates (10). Most validations use the same multiple of the median (MoM) equation for the calculation of expected values for each biomarker used for risk classification, which is an equation of how the distribution of the biomarker influenced the population’s characteristics. Medical history and anthropometric features have demonstrated a significant influence on each biomarker’s distribution, and this distribution depends on the characteristics of each population. An example of this is the demonstrated increased risk of preeclampsia in patients that underwent IVF treatments in case of freezing eggs after ovarian stimulation (11). It is then logical to think that failure to achieve similar detection rates to that of the original Fetal Medicine Foundation (FMF) model may arise from the lack of original MoMs created in each population, as differences in disease prevalence and anthropometric characteristics from population to population may significantly alter the distribution of each biomarker. Therefore, we hypothesize that the maternal characteristics of our population have a unique influence on the distribution of each biomarker different from that of the original FMF model.

Thus, the objective of this study is to identify and quantify the effects of maternal characteristics and medical history on the distribution of PlGF, MAP, and UtA-PI; and to standardize the expected values for these biomarkers in the first trimester to create unique MoMs for Latin-American population.

## Materials and methods

### Study design and participants

This is a prospective cohort of consecutive pregnant women attending their first-trimester screening ultrasound at a primary care center for the general population in Mexico City between April 2019 and October 2021.

Criteria for inclusion were pregnant women aged 18 years or older with a singleton pregnancy recruited at their first-trimester scan. Exclusion criteria were chromosomal abnormalities, major fetal malformations, and women delivering in another care center. The cohort was built exclusively for research purposes to ensure maximum quality on data acquirement and measurement. Ultrasounds were performed by two maternal-fetal medicine specialists with an FMF certification for first-trimester screening for preeclampsia. The protocol was approved by the Ethics and Research Internal Review Board of the National Institute of Perinatology (2021-1-38) and was conducted ethically under the World Medical Association Declaration of Helsinki. All enrolled women authorized its inclusion and provided signed informed consent.

### Data collection

Data was acquired from secured electronic medical records. Ultrasounds were performed at first trimester screening for chromosomal abnormalities and preeclampsia (between 11 and 13 + 6 weeks). Maternal characteristics included those required by the FMF preeclampsia screening program, such as maternal age, spontaneous pregnancy or use of assisted reproduction techniques; parity (nulliparous or parous previous), cigarette smoking, drugs addiction (cocaine or heroin), alcohol intake during pregnancy, mother with PE, history of PE in previous pregnancy, fetal growth restriction in previous pregnancy, gestational diabetes, chronic hypertension, diabetes mellitus, systemic lupus erythematosus (SLE), antiphospholipid syndrome (APS). Additional information on maternal heart disease, polycystic ovary syndrome (PCOS), and hypothyroidism were included. Anthropometric biomarkers included: height, weight, body mass index (BMI), mean arterial pressure (MAP), and mean uterine artery pulsatility index (mUtA-PI). The serum biomarker was PlGF, and the blood samples for PlGF measurements were also drawn at the first trimester ultrasound.

Gestational age was determined from the measurement of the fetal crown-rum length. MAP and UtA-PI were measured according to standardized protocols by certified maternal-fetal medicine specialists (7, 12). Maternal serum concentrations of PlGF (Elecsys PlGF, Roche^®^) were measured using an automated analyzer (Cobas-e411, Roche^®^) according to the manufacturer’s instructions.

### Outcome measures

Data on pregnancy outcomes were collected from hospital records. PE was defined according to American College of Obstetricians and Gynecologists (ACOG) criteria (13): hypertension (systolic blood pressure of ≥140 mmHg or diastolic blood pressure of ≥90 mmHg) developed after 20 weeks of gestation in previously normotensive women and at least one of the following: proteinuria (≥300 mg/24 h), renal insufficiency (serum creatinine ≥1.1 mg/dL or twofold increase in serum creatinine in the absence of underlying renal disease), liver involvement (blood concentration of transaminases to twice the normal level), neurological complications (cerebral o visual symptoms), or thrombocytopenia (platelet count < 100,000 U/L). Early preeclampsia (ePE) was defined as those presented before 34 weeks of gestation, preterm preeclampsia (pPE) was defined as PE that occurred before 37 weeks of gestation, and term preeclampsia (tPE) as PE occurred after 37 weeks of gestation.

### Statistical analysis

First-trimester PlGF, MAP, and mUtA-PI values were log-transformed to normalize their distribution. The expected values for non-preeclamptic women were created by assessing the influence of maternal characteristics on each log-transformed biomarker in multiple linear regression. Backward elimination was used to identify potentially important terms in the model by sequentially removing non-significant (*P* > 0.05) variables. The effect sizes were assessed considering the standard deviation (SD), and a criterion of 0.1 SD was used to exclude terms that lack substantive impact on model predictions. Predicted model for statistically significant variables was used as the expected values. Multiples of the median (MoM) were created by the difference between the observed and expected values for all patients. The observed MoM value was divided by the expected value based on regression analysis of the controls, normalizing each observation. We also calculated the exponential of the standardized difference between the observed concentration and the population median for the gestational age divided by his standard error. Residual analyzes were used to assess the suitability of the model. Graphical displays of the relationship between head-rump length, maternal age, biomarkers, and the effects of maternal characteristics and medical history on the PlGF MoM, MAP MoM, and UAt-PI MoM values were produced for the final model (StataCorp., 2020, Stata Statistical Software: Release 17. College Station, TX: StataCorp LLC).

## Results

### Description of the cohort and characteristics of the study population

A total of 3,067 pregnant women were enrolled in the original cohort, and 247 women (8.05%) were excluded due to incomplete data. Among 2,820 pregnant women included in the final analysis, 118 (4.18%) developed PE, of which 22 (0.78%) were delivered before 34 weeks of gestation (ePE), 74 (2.62%) developed pPE, and 44 (1.56%) developed tPE. Table 1 describes the characteristics of the population by outcome measures.

**TABLE 1:** Characteristics of the included population.

Characteristic*	Control group *n* = 2702	Early PE *n* = 22	Preterm PE *n* = 52	Term PE *n* = 44	*p*-value
Maternal age (years)	28.71	35.11	30.64	32.47	0.0001
	(22.73–34.19)	(27.86–39.08)	(23.45–37.19)	(24.99–36.71)	
Nulliparity	928 (34.34%)	10 (45.45%)	22 (42.31%)	10 (22.73%)	0.152
Spontaneous pregnancy	2657 (98.34%)	22 (100%)	52 (100%)	44 (100%)	0.452
Induction of ovulation	26 (0.96%)	0.0 (%)	0.0 (%)	0.0 (%)	0.766
IVF	19 (0.70%)	0.0 (%)	0.0 (%)	0.0 (%)	0.841
Gestational age at screening	12.73	13.12	12.67	12.65	0.125
	(12.26–13.17)	(12.65–13.48)	(12.24–12.94)	(12.10–13.45)	
Smoker	178 (6.59%)	0.0 (%)	3 (5.77%)	4 (9.09%)	0.560
Alcohol intake	49 (1.81%)	0.0 (%)	1 (1.92%)	0.0 (%)	0.771
Other drugs	49 (1.81%)	0.0 (%)	2 (3.85%)	0.0 (%)	0.488
Pre-existing diabetes	136 (5.03%)	3 (13.64%)	6 (11.54%)	0.0 (%)	0.018
Chronic hypertension	84 (3.11%)	3 (13.64%)	12 (23.08%)	0.0 (%)	< 0.000
SLE	22 (0.81%)	1 (4.55%)	0.0 (%)	0.0 (%)	0.206
Antiphospholipid syndrome	9 (0.33%)	3 (13.64%)	4 (7.69%)	0.0 (%)	0.000
Polycystic ovary syndrome	65 (2.42%)	0.0 (%)	3 (5.77%)	1 (2.38%)	0.402
Hypothyroidism	252 (9.33%)	0.0 (%)	2 (3.85%)	0.0 (%)	0.036
Congenital cardiac disease	38 (1.41%)	0.0 (%)	0.0 (%)	0.0 (%)	0.641
PE in a previous pregnancy	171 (6.33%)	7 (31.82%)	9 (17.31%)	10 (22.73%)	0.000
FGR in previous pregnancy	175 (6.48%)	1 (4.55%)	5 (9.62%)	0	0.256
Gestational diabetes in a previous pregnancy	36 (1.33%)	0.0 (%)	5 (9.62%)	1 (2.27%)	< 0.000
Mother of the patient had PE	142 (5.26%)	0.0 (%)	6 (11.54%)	0.0 (%)	0.050
BMI	26.08	28.44	29.16	31.86	0.0001
	(23.18–29.47)	(22.43–30.65)	(26.55–33.25)	(24.77–36.27)	
MAP (mmHg)	75.58	86.17	85.38	83.66	0.0001
	(70.25–81.25)	(83.83–91.33)	(79.69–89.67)	(74.83–85.63)	
Fetal CRL (mm)	64.95	70.8	64.1	63.7	0.125
	(58.3–71.6)	(63.8–76.4)	(57.9–68.1)	(56.05–76.0)	
UtA-PI	1.5	2.58	1.92	1.53	0.0001
	(1.21–1.89)	(1.85–3.14)	(1.58–2.36)	(1.17–1.68)	
PlGF (pg/ml)	23.65	14.88	14.82	21.5	0.0001
	(17.42–32.8)	(12.14–17.61)	(10.09–18.8)	(18.86–25.84)	
Gestational age at delivery	38.5	33.3	36.4	38.2	0.0001
	(37.4–39.4)	(33.2–33.6)	(35.4–36.6)	(37.5–39.1)	

PE, preeclampsia; IVF, *in vitro* fertilization; FGR, fetal growth restriction; SLE, systemic lupus erythematosus; BMI, body mass index; MAP, mean arterial pressure; CRL, crown-rump length; UtA-PI, uterine artery pulsatility index; PlGF, placental growth factor. *All characteristics were measured during the first trimester screening for aneuploidies (11–13 + 6 weeks).

### Maternal characteristics influencing first trimester biomarkers

**FIGURE 1:**
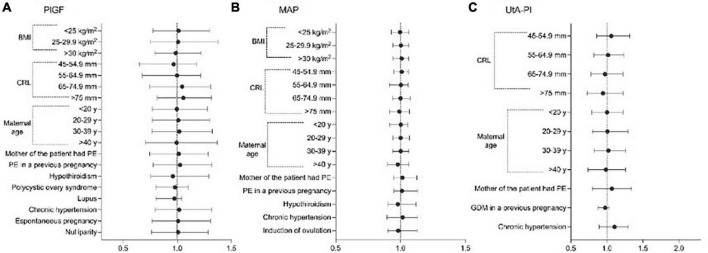
Significant variables affecting the distribution of Log values of; **(A)** placental growth factor (PlGF), **(B)** mean arterial pressure (MAP), and **(C)** uterine artery pulsatility index (UtA-PI), plotted on multiples of the median (MoM) scale among healthy pregnancies.

After multiple regression analysis, independent contributions for the prediction of log_10_PlGF, log_10_MAP, and log_10_UAt-PI in the first trimester are shown in Table 2. The values of MoMs for PlGF, MAP, and UAt-PI are presented in Supplementary Tables 1–3 and Figure 1. Supplementary Tables 4–6 contain the values of PlGF, MAP, and UAt-PI MoM by categories of maternal characteristics and medical history in pregnant women with ePE, pPE, lPE, and without PE.

**TABLE 2:** Multiple regression analysis to identify significant independent contributors to first trimester biomarkers.

Variable*	Estimate	95% CI	*p*-value
PlGF			
Fetal CRL	0.0191399	0.0174415 to 0.0208382	<0.000
Maternal age	0.0028178	0.0004532 to 0.0051824	<0.020
Nulliparity	-0.0464928	−0.0812951 to −0.0116905	0.009
BMI	-0.011891	−0.015176 to −0.008606	<0.000
Chronic hypertension	-0.1000511	−0.1931976 to −0.0069047	0.035
SLE	-0.3163534	−0.4892293 to −0.1434775	<0.000
Spontaneous pregnancy	0.2036425	0.0962791 to 0.3110059	<0.000
PCOS	-0.1206829	−0.223592 to −0.0177738	0.022
Hypothyroidism	-0.1066501	−0.160929 to −0.0523713	<0.000
PE in a previous pregnancy	-0.1111554	−0.1778724 to −0.0444383	0.001
History of PE in the mother of patient	-0.0915202	−0.1617922 to −0.0212482	0.011
**MAP**			
Fetal CRL	-0.0008	−0.0012129 to −0.0003871	<0.000
Maternal age	0.0010257	0.0004652 to 0.0015862	<0.000
PE in a previous pregnancy	0.0290743	0.0130468 to 0.0451018	<0.000
Induction of ovulation	-0.0569733	−0.0964335 to −0.0175132	<0.005
Mother of the patient had PE	0.044145	0.0270095 to 0.0612806	<0.000
Chronic hypertension	0.0499594	0.0272924 to 0.0726265	<0.000
BMI	0.0065639	0.0057692 to 0.0073585	<0.000
Hypothyroidism	-0.0232769	−0.0364032 to −0.0101506	0.001
**UtA-PI**			
Fetal CRL	-0.0059239	−0.0073045 to −0.0045432	<0.000
Maternal age	-0.0023316	−0.0041588 to −0.0005044	0.012
Mother of the patient had PE	0.1409438	0.0840537 to 0.1978339	<0.000
Chronic hypertension	0.0750184	0.00046 to 0.1495769	0.049
GDM in previous pregnancy	-0.211569	−0.3235432 to −0.0995948	<0.000

PE, preeclampsia; IVF, *in vitro* fertilization; FGR, fetal growth restriction; SLE, systemic lupus erythematosus; BMI, body mass index; MAP, mean arterial pressure; CRL, crown-rump length; UtA-PI, uterine artery pulsatility index; PlGF, placental growth factor. *All variables were measured during the first trimester screening for aneuploidies (11–13 + 6 weeks).

### Distributional properties of the MoM values among non-pregnant women

**FIGURE 2:**
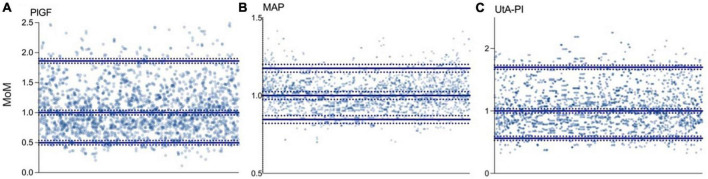
Distribution of biomarkers in the control group. **(A)** Placental growth factor (PlGF): median 1.000 (95% CI, 0.989–1.024), p5 0.498 (95% CI, 0.481–0.514), and p95 1.861 (95% CI, 1.843–1.877). **(B)** Mean arterial pressure (MAP): median 1.000 (95% CI, 0.962–1.039), p5 0.846 (95% CI, 0.807–0.884), and p95 1.175 (95% CI, 1.136–1.213). **(C)** Uterine artery pulsatility index (UtA-PI): median 1.000 (95% CI, 0.996–1.021), p5 0.561 (95% CI, 0.547–0.574), and p95 1.699 (95% CI, 1.685–1.712).

Multiples of the median values of PlGF, MAP, and UtA-PI in the control group during the first- trimester are shown in Figure 2. The median, 5th, and 95th percentiles of PlGF were 1.000 (95% CI, 0.989–1.024), 0.498 (95% CI, 0.481–0.514), and 1.861 (95% CI, 1.843–1.877), respectively. For MAP, median, 5th, and 95th percentiles were 1.000 (95% CI, 0.962–1.039), 0.846 (95% CI, 0.807–0.884), and 1.175 (95% CI, 1.136–1.213). The median, 5th, and 95th percentiles for UAt-PI were 1.000 (95% CI, 0.996–1.021), 0.561 (95% CI, 0.547–0.574), and 1.699 (95% CI, 1.685–1.712).

### Comparison of first-trimester PlGF, MAP, and UtA-PA MoMs between controls and PE

**FIGURE 3:**
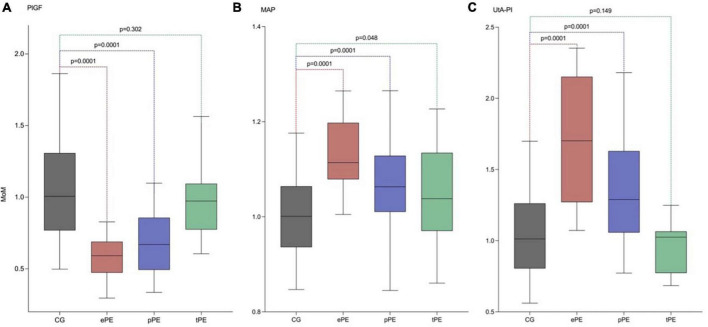
Adjusted median, p5, and p95 of PlGF, MAP, and UtA-PI in the control group (CG), early preeclampsia (ePE), preterm preeclampsia (pPE), and term preeclampsia (tPE) using our model of maternal characteristics and medical history. **(A)** Serum placental growth factor (PlGF). **(B)** Values of MoM of the mean arterial pressure (MAP). **(C)** Values of MoM of the uterine artery pulsatility index (UtA-PI).

There was a significant difference among MoMs of the different biomarkers between controls and women developing early, late, and term preeclampsia. There was a significant difference in lower MoMs for PlGF among ePE that increased among pPE, but there was no difference between controls and tPE. However, for MAP MoMs, values were higher among ePE and were lower for pPE and tPE; there was a significant difference in MAP MoMs among all women developing PE and controls. Regarding UtA-PI MoMs, there was a significant difference in higher MoMs among ePE and pPE but not of tPE compared to controls (Figure 3). Table 3 shows the median and 95% CI for each biomarker MoM according to PE status.

**TABLE 3:** Median, p5, and p95 of MoM according to PE status.

Biomarker*	Control group			Early preeclampsia			Preterm preeclampsia			Term preeclampsia		
	Median	p5	p95	Median	p5	p95	Median	p5	p95	Median	p5	p95
	(95% CI)	(95% CI)	(95% CI)	(95% CI)	(95% CI)	(95% CI)	(95% CI)	(95% CI)	(95% CI)	(95% CI)	(95% CI)	(95% CI)
PlGF	1.00	0.498	1.861	0.592	0.325	0.809	0.669 (0.609–0.740)	0.339	1.069	0.973 (0.893–1.054)	0.607	1.440
	(0.989–1.024)	(0.481–0.514)	(1.843–1.877)	(0.519–0.652)	(0.259–0.391)	(0.743–0.875)		(0.276–0.402)	(1.006–1.132)		(0.529–0.685)	(1.362–1.518)
MAP	1.00	0.846	1.175	1.114	1.049	1.262	1.063 (1.037–1.096)	0.852	1.261	1.038 (1.008–1.073)	0.902	1.208
	(0.962–1.039)	(0.807–0.884)	(1.136–1.213)	(1.105–1.173)	(1.015–1.082)	(1.228–1.295)		(0.823–0.880)	(1.232–1.289)		(0.871–0.933)	(1.177–1.239)
UtA-PI	1.00	0.561	1.699	1.702	1.099	2.285	1.289 (1.264–1.499)	0.781	2.177	1.025 (0.922–1.043)	0.706	1.246
	(0.996–1.021)	(0.547–0.574)	(1.685–1.712)	(1.529–1.932)	(0.898–1.133)	(2.084–2.486)		(0.670–0.899)	(2.062–2.291)		(0.647–0.765)	(1.187–1.304)

p, percentile; PlGF, placental growth factor; MAP, mean arterial pressure; UtA-PI, uterine artery pulsatility index; CI, confidence interval. *All biomarkers were measured during the first trimester screening for aneuploidies (11–13 + 6 weeks).

## Discussion

### Main findings

The findings of this study are first that there are maternal characteristics that significantly influence the distribution of the Log values for PlGF, MAP, and UtA-PI that are similar to other studies and some others that are unique for our population, such as PCOS, hypothyroidism, and gestational diabetes mellitus (GDM) in previous pregnancies. Second, the creation of MoMs using these new distributions can discriminate between women developing PE later in pregnancy with a trend toward more extreme values among ePE compared to controls.

### Comparison with existing literature

Extensive studies in screening for preeclampsia have established that all biomarkers used for the prediction of this disease should be expressed as MoMs after adjustment for maternal characteristics in normal pregnancies (14–17). However, the employment of non-population-specific models for MoM calculation leads to an imprecise prediction of PE (18–21). We found that PlGF, MAP, and UtA-PI’s distribution are influenced by several maternal characteristics that are similar to other populations, such as the example of reproductive assisted techniques (11), anthropometric features, age, and crown rump length (CRL). However, there are other unique population features that have not been described in previous studies, such as PCOS. In this study, we found that pregnant women with a history of PCOS that did not develop preeclampsia have lower PlGF levels. An explanation of this may be the prevalence of hypothyroidism in females, which varies between 0.3 and 3.7% in the USA and between 0.2 and 6.4% in Europe (22–24). In Mexico, the prevalence of PCOS is 8% (25), and our data shows a prevalence of 9%, which emphasizes the importance of this disease in our population due to its high prevalence and the need for reproductive techniques that arise in these women that may appear as a confounder along PCOS.

Maternal hypothyroidism is another disease that has a higher prevalence in Latin America; this disease can lead to low levels of placental T4 and T3, resulting in a decreased expression of PlGF (26). We have observed lower PlGF MoMs in pregnant women with hypothyroidism that did not develop preeclampsia which goes hand to hand with a recent meta-analysis that showed that higher thyroid stimulating hormone (TSH) levels were associated with a higher risk of preeclampsia (27). Even though we did not find significant differences in the prevalence of hypothyroidism among the control group and preeclampsia groups, most patients are under control using levothyroxine therapy which has shown a decrease in MAP that is coherent with other studies that show a blood pressure-lowering effect of levothyroxine therapy in hypothyroidism (28, 29).

Another interesting finding of this study is that GDM influences UtA-PI among women who did not develop preeclampsia; this has also been supported by Sweeting et al. showing that GDM was associated with lower values of UtA-PI MoMs (30). However, there is no clear explanation of how GDM may directly influence placentation and UtA-PI values. However, evidence is consistent by showing the same results in other studies as previously described.

Our study shows that maternal characteristics and diseases such as PCOS, hypothyroidism, and GDM are more prevalent in our population and that these characteristics affect the distribution of the Log values of PlGF, MAP, and UtA-PI in women that did not develop preeclampsia. It is then coherent that the development of new expected values for these biomarkers is justified for our population, given the significant effect of diseases and maternal characteristics that are not present in Europeans or other populations.

### Strengths and limitations

The strengths of our study were that pregnant women were consecutively recruited in a study with a specialized database built specifically for research purposes, minimizing potential biases; the use of a validated methodology and automated devices by trained doctors to measure PlGF, MAP, and UtA-IP; and application of multiple regression analysis to define the contributions and interrelationships of maternal variables that influence measured each biomarker in the first trimester. But as we have strengths, we also have limitations. Our main limitation is the small sample of ePE; nevertheless, for MoM creation, the importance lies in the control group from which we create the expected values for healthy pregnancies. Another limitation is the lack of severe COVID-19 as a risk factor in maternal history since COVID is an endothelial disease that could potentially have an increased risk on the development of PE in subsequent pregnancies. Also, it may be argued that there may be a confounding among several characteristics such as PCOS, assisted reproductive techniques, and PE; nevertheless, we used a multiple regression analysis to adjust for confounders to find which characteristics are significantly independent and how much they influence the distribution of all biomarkers in healthy women.

### Clinical interpretation

Identifying population-specific characteristics that influence the distribution of PlGF, MAP, and UtA-PI in healthy pregnancies is the first step in creating new predictive models for PE in our region. Nevertheless, it is also possible that these new MoMs could help improve existing predictive models to help increase their detection rate for PE. In the end, under the law of parsimoniousness, it is not valid to aim for the most statistically complex or advance model as if we were competing for who develops the hardest prediction model; the simplest model with the better prediction is the one that we should use in our population.

## Conclusion

Several maternal characteristics influence the expected log values for PlGf, MAP, and UtA-PI that are similar to other populations and some other features that are specific to our region, such as PCOS, hypothyroidism, and GDM. The development of population-specific MoMs can discriminate among women that will develop PE during pregnancy.

## Data availability statement

The raw data supporting the conclusions of this article will be made available by the authors upon a reasonable request.

## Ethics statement

This study was reviewed and approved by the Ethics and Research Internal Review Board of the National Institute of Perinatology. The patients/participants always provided their written informed consent to participate in this study.

## Author contributions

JT-T conceived the idea and helped draft the manuscript. SE-y-S helped draft the manuscript. JV-B performed a critical review and helped in the statistical analysis. LO-G helped in the statistical analysis. JS-P, GE-G, RM-C, and SA-G performed a critical review. PM-R helped draft the manuscript. RM-P conceived the idea, helped draft the manuscript, and performed the critical review and statistical analysis. All authors contributed to the article and approved the submitted version.
