# Supplementary material for: Effects of maternal characteristics and medical history on first trimester biomarkers for preeclampsia
Source: Front Med (Lausanne). 2023 Jan 24;10:1050923. doi: 10.3389/fmed.2023.1050923 (PMC9902506; doi:10.3389/fmed.2023.1050923)
Supplement: Supplementary file 1 [file Table_1.DOCX]

| **Table S1.** MoM of placental growth factor (PlGF) by categories of independent predictors in control group | | | |
| --- | --- | --- | --- |
| Variable | Multiples of the Median (MoM) | | |
|  | Median | IQR | |
| Maternal age |  |  |  |
| >40 years | 0.99 | 1.37 | 0.71 |
| 30-39 years | 1.01 | 1.32 | 0.77 |
| 20-29 years | 1.01 | 1.31 | 0.77 |
| <20 years | 0.99 | 1.27 | 0.77 |
| Fetal crown-rum length |  |  |  |
| >75 mm | 1.05 | 1.32 | 0.82 |
| 65-74.9 mm | 1.04 | 1.31 | 0.75 |
| 55-64.9 mm | 0.99 | 1.22 | 0.68 |
| 45-54.9 mm | 0.97 | 1.18 | 0.65 |
| Body mass index |  |  |  |
| >30 kg/m^2^ | 0.98 | 1.22 | 0.79 |
| 25-29.9 kg/m^2^ | 1.01 | 1.38 | 0.75 |
| <25 kg/m^2^ | 1.01 | 1.30 | 0.78 |
| Nulliparity | 1.01 | 1.29 | 0.76 |
| Spontaneous pregnancy | 1.01 | 1.31 | 0.77 |
| Chronic hypertension | 1.02 | 1.32 | 0.8 |
| Lupus | 0.97 | 1.04 | 0.8 |
| Polycystic ovary syndrome | 0.98 | 1.04 | 0.8 |
| Hypothiroidism | 0.98 | 1.28 | 0.76 |
| PE in a previous pregnancy | 1.02 | 1.32 | 0.78 |
| Mother of the patient had PE | 1.01 | 1.29 | 0.74 |
| IQR: interquartile range; PE: preeclampsia | | | |

|  | | | |
| --- | --- | --- | --- |
| **Table S2.** MoM of mean arterial pressure (MAP) by categories of independent predictors in control group | | | |
| Variable | Multiples of the Median (MoM) | | |
|  | Median | IQR | |
| Maternal age |  |  |  |
| >40 years | 0.98 | 1.07 | 0.90 |
| 30-39 years | 1.00 | 1.06 | 0.94 |
| 20-29 years | 1.00 | 1.06 | 0.94 |
| <20 years | 1.01 | 1.05 | 0.91 |
| Fetal crown-rum length |  |  |  |
| >75 mm | 0.99 | 1.07 | 0.91 |
| 65-74.9 mm | 1.00 | 1.08 | 0.94 |
| 55-64.9 mm | 1.00 | 1.06 | 0.91 |
| 45-54.9 mm | 1.01 | 1.06 | 0.95 |
| Body mass index |  |  |  |
| >30 kg/m^2^ | 1.01 | 1.06 | 0.94 |
| 25-29.9 kg/m^2^ | 1.00 | 1.06 | 0.94 |
| <25 kg/m^2^ | 0.99 | 1.06 | 0.93 |
| Induction of ovulation | 0.99 | 1.13 | 0.90 |
| Chronic hypertension | 1.01 | 1.13 | 0.89 |
| Hypothiroidism | 0.96 | 1.12 | 0.91 |
| PE in a previous pregnancy | 1.01 | 1.13 | 0.95 |
| Mother of the patient had PE | 1.01 | 1.12 | 0.94 |
| IQR: interquartile range; PE: preeclampsia | | | |

| **Table S3.** MoM of uterine artery pulsatility index (UtA-PI) by categories of independent predictors in control group | | | |
| --- | --- | --- | --- |
| Variable | Multiples of the Median (MoM) | | |
|  | Median | IQR | |
| Maternal age |  |  |  |
| >40 years | 0.99 | 1.26 | 0.74 |
| 30-39 years | 1.02 | 1.26 | 0.82 |
| 20-29 years | 1.00 | 1.29 | 0.80 |
| <20 years | 1.00 | 1.22 | 0.79 |
| Fetal crown-rum length |  |  |  |
| >75 mm | 0.99 | 1.23 | 0.74 |
| 65-74.9 mm | 0.98 | 1.22 | 0.78 |
| 55-64.9 mm | 1.02 | 1.23 | 0.82 |
| 45-54.9 mm | 1.06 | 1.32 | 0.86 |
| Chronic hypertension | 1.10 | 1.29 | 0.89 |
| Mother of the patient had PE | 1.07 | 1.34 | 0.80 |
| GDM in a previous pregnancy | 0.98 | 1.03 | 0.88 |
| IQR: interquartile range; PE: preeclampsia; GDM: gestational diabetes mellitus | | | |

| **Table S4.** Values of PlGF MoM by categories of maternal characteristics and medical history among pregnant women with and without PE | | | | | | | | | | | | |
| --- | --- | --- | --- | --- | --- | --- | --- | --- | --- | --- | --- | --- |
| Variable | Placental growth factor (PlGF) | | | | | | | | | | | |
|  | Control group | | | Early Preeclampsia | | | Preterm Preeclampsia | | | Late Preeclampsia | | |
|  | MoM | | | MoM | | | MoM | | | MoM | | |
|  | Median | IQR | | Median | IQR | | Median | IQR | | Median | IQR | |
| Nulliparity | 1.01 | 1.29 | 0.76 | 0.56 | 0.59 | 0.48 | 0.59 | 0.92 | 0.49 | 1.13 | 1.45 | 0.89 |
| Spontaneous pregnancy | 1.01 | 1.31 | 0.77 | 0.58 | 0.68 | 0.52 | 0.59 | 0.89 | 0.48 | 0.94 | 1.05 | 0.72 |
| Chronic hypertension | 1.02 | 1.3 | 0.8 | 0.59 | 0.77 | 0.59 | 0.78 | 1.25 | 0.56 | --- | --- | --- |
| SLE | 0.99 | 1.03 | 0.8 | 0.65 | 0.65 | 0.65 | --- | --- | --- | --- | --- | --- |
| Polycystic ovary syndrome | 0.98 | 1.14 | 0.8 | --- | --- | --- | 0.82 | 1.05 | 0.58 | 0.84 | 0.84 | 0.84 |
| Hypothiroidism | 0.99 | 1.28 | 0.76 | --- | --- | --- | 0.63 | 0.67 | 0.59 | --- | --- | --- |
| PE in a previous pregnancy | 1.02 | 1.33 | 0.78 | 0.523 | 0.65 | 0.45 | 0.69 | 0.88 | 0.67 | 1.206372 | 1.670266 | 0.9373791 |
| Mother of the patient had PE | 1.02 | 1.29 | 0.75 | --- | --- | --- | 0.45 | 0.67 | 0.39 | --- | --- | --- |
| Maternal age |  |  |  |  |  |  |  |  |  |  |  |  |
| >40 years | 0.99 | 1.37 | 0.70 | 0.52 | 0.66 | 0.44 | 0.52 | 0.66 | 0.39 | 0.73 | 1.04 | 0.69 |
| 30-39 years | 1.01 | 1.32 | 0.76 | 0.61 | 0.73 | 0.46 | 0.57 | 0.69 | 0.48 | 0.84 | 0.84 | 0.84 |
| 20-29 years | 1.01 | 1.30 | 0.76 | 0.59 | 0.65 | 0.54 | 0.62 | 0.92 | 0.54 | 0.94 | 0.94 | 0.94 |
| <20 years | 0.99 | 1.27 | 0.77 | --- | --- | --- | 0.50 | 0.69 | 0.37 | 1.01 | 1.02 | 0.95 |
| Fetal crown-rum length |  |  |  |  |  |  |  |  |  |  |  |  |
| >75 mm | 1.05 | 1.31 | 0.81 | 0.54 | 0.57 | 0.33 | 0.55 | 0.62 | 0.42 | 0.75 | 0.94 | 0.71 |
| 65-74.9 mm | 1.04 | 1.31 | 0.74 | 0.59 | 0.66 | 0.52 | 0.59 | 1.02 | 0.52 | 1.06 | 1.08 | 0.95 |
| 55-64.9 mm | 0.99 | 1.22 | 0.68 | 0.68 | 0.68 | 0.68 | 0.49 | 0.69 | 0.38 | 0.89 | 1.01 | 0.60 |
| 45-54.9 mm | 0.95 | 1.18 | 0.65 | 0.77 | 0.83 | 0.69 | 0.69 | 0.79 | 0.49 | 1.01 | 1.02 | 1.00 |
| Body mass index |  |  |  |  |  |  |  |  |  |  |  |  |
| >30 kg/m^2^ | 0.98 | 1.22 | 0.79 | 0.52 | 0.66 | 0.45 | 0.58 | 0.62 | 0.54 | 0.82 | 1.01 | 0.71 |
| 25-29.9 kg/m^2^ | 1.01 | 1.38 | 0.75 | 0.57 | 0.59 | 0.54 | 0.59 | 0.77 | 0.37 | 1.06 | 1.08 | 0.84 |
| <25 kg/m^2^ | 1.01 | 1.30 | 0.78 | 0.66 | 0.73 | 0.45 | 0.59 | 0.89 | 0.49 | 1.01 | 1.02 | 0.83 |
|  | | | | | | | | | | | | |

| **Table S5**. Values of MAP MoM by categories of maternal characteristics and medical history among pregnant women with and without PE | | | | | | | | | | | | |
| --- | --- | --- | --- | --- | --- | --- | --- | --- | --- | --- | --- | --- |
| Variable | Mean arterial pressure (MAP) | | | | | | | | | | | |
|  | Control group | | | Early Preeclampsia | | | Preterm Preeclampsia | | | Late Preeclampsia | | |
|  | MoM | | | MoM | | | MoM | | | MoM | | |
|  | Median | IQR | | Median | IQR | | Median | IQR | | Median | IQR | |
| Induction of ovulation | 1.03 | 0.91 | 1.03 | --- | --- | --- | --- | --- | --- | --- | --- | --- |
| Chronic hypertension | 1.04 | 0.95 | 1.04 | 1.26 | 1.29 | 1.24 | 1.15 | 1.19 | 1.04 | --- | --- | --- |
| Hypothiroidism | 1.08 | 0.93 | 1.08 | --- | --- | --- | 1.18 | 1.23 | 1.13 | --- | --- | --- |
| PE in a previous pregnancy | 1.06 | 0.93 | 1.06 | 1.07 | 1.19 | 1.06 | 1.11 | 1.18 | 0.92 | 0.96 | 1.04 | 0.91 |
| Mother of the patient had PE | 1.06 | 0.95 | 1.06 | --- | --- | --- | 1.01 | 1.10 | 0.99 | --- | --- | --- |
| Maternal age |  |  |  |  |  |  |  |  |  | --- | --- | --- |
| >40 years | 0.98 | 1.07 | 0.89 | 1.15 | 1.19 | 1.09 | 1.15 | 1.18 | 0.99 | 1.08 | 1.08 | 1.08 |
| 30-39 years | 1.01 | 1.06 | 0.94 | 1.11 | 1.19 | 1.06 | 1.10 | 1.11 | 1.01 | 1.02 | 1.04 | 0.94 |
| 20-29 years | 1.01 | 1.07 | 0.94 | 1.11 | 1.26 | 1.06 | 1.09 | 1.14 | 1.05 | 1.07 | 1.15 | 0.99 |
| <20 years | 1.01 | 1.05 | 0.91 | --- | --- | --- | 1.06 | 1.08 | 1.04 | 0.85 | 0.85 | 0.85 |
| Fetal crown-rum length |  |  |  |  |  |  |  |  |  |  |  |  |
| >75 mm | 0.99 | 1.07 | 0.91 | 1.11 | 1.19 | 1.06 | 1.08 | 1.10 | 0.99 | 1.03 | 1.04 | 0.99 |
| 65-74.9 mm | 1.01 | 1.08 | 0.94 | 1.11 | 1.19 | 1.10 | 1.09 | 1.11 | 1.05 | 0.91 | 0.91 | 0.90 |
| 55-64.9 mm | 1.01 | 1.06 | 0.91 | 1.14 | 1.25 | 1.08 | 1.10 | 1.12 | 1.05 | 1.06 | 1.13 | 1.01 |
| 45-54.9 mm | 1.01 | 1.06 | 0.95 | 1.17 | 1.17 | 1.17 | 1.15 | 1.22 | 1.07 | 1.16 | 1.16 | 1.15 |
| Body mass index |  |  |  |  |  |  |  |  |  |  |  |  |
| >30 kg/m^2^ | 1.01 | 1.06 | 0.94 | 1.14 | 1.26 | 1.11 | 1.14 | 1.26 | 1.11 | 1.15 | 1.16 | 1.13 |
| 25-29.9 kg/m^2^ | 1.01 | 1.06 | 0.94 | 1.13 | 1.19 | 1.07 | 1.08 | 1.10 | 0.99 | 0.92 | 0.99 | 0.91 |
| <25 kg/m^2^ | 0.99 | 1.06 | 0.93 | 1.11 | 1.19 | 1.06 | 1.08 | 1.11 | 1.05 | 1.03 | 1.05 | 1.01 |
|  | | | | | | | | | | | | |

| **Table S6.** Values of UtA-PI MoM by categories of maternal characteristics and medical history among pregnant women with and without PE | | | | | | | | | | | | |
| --- | --- | --- | --- | --- | --- | --- | --- | --- | --- | --- | --- | --- |
| Variable | Uterine artery pulsatility index (UtA-PI) | | | | | | | | | | | |
|  | Control group | | | Early Preeclampsia | | | Preterm Preeclampsia | | | Late Preeclampsia | | |
|  | MoM | | | MoM | | | MoM | | | MoM | | |
|  | Median | IQR | | Median | IQR | | Median | IQR | | Median | IQR | |
| Chronic hypertension | 1.01 | 1.26 | 0.8 | 2.26 | 2.29 | 1.69 | 1.27 | 1.6 | 0.89 | --- | --- | --- |
| Mother of the patient had PE | 1.01 | 1.26 | 0.8 | --- | --- | --- | 1.08 | 1.10 | 0.99 | 1.29 | 1.29 | 1.29 |
| GDM in a previous pregnancy | 0.88 | 0.96 | 0.73 | --- | --- | --- | 1.28 | 1.70 | 0.99 | --- | --- | --- |
| Maternal age |  |  |  |  |  |  |  |  |  |  |  |  |
| >40 years | 0.99 | 1.26 | 0.74 | 2.26 | 2.29 | 1.21 | 1.58 | 2.18 | 1.10 | 1.24 | 1.24 | 1.24 |
| 30-39 years | 1.02 | 1.26 | 0.82 | 1.73 | 2.11 | 1.43 | 1.46 | 1.99 | 1.10 | 0.92 | 1.06 | 0.75 |
| 20-29 years | 1.01 | 1.29 | 0.79 | 1.51 | 2.27 | 0.99 | 1.28 | 1.57 | 0.98 | 1.03 | 1.06 | 0.90 |
| <20 years | 1.01 | 1.22 | 0.79 | --- | --- | --- | 1.70 | 1.96 | 1.07 | 1.05 | 1.05 | 1.05 |
| Fetal crown-rum length |  |  |  |  |  |  |  |  |  |  |  |  |
| >75 mm | 0.95 | 1.23 | 0.73 | 1.69 | 2.15 | 0.99 | 1.27 | 1.87 | 0.98 | 0.87 | 0.97 | 0.83 |
| 65-74.9 mm | 0.97 | 1.23 | 0.78 | 2.27 | 2.27 | 1.10 | 1.23 | 2.13 | 0.98 | 1.06 | 1.06 | 0.85 |
| 55-64.9 mm | 1.01 | 1.23 | 0.82 | 1.63 | 1.69 | 1.24 | 1.58 | 1.88 | 1.18 | 1.04 | 1.16 | 1.02 |
| 45-54.9 mm | 1.06 | 1.32 | 0.86 | 2.07 | 2.07 | 2.07 | 1.37 | 1.71 | 1.17 | 1.04 | 1.04 | 0.84 |
|  | | | | | | | | | | | | |
